# Distribution patterns of foot and ankle tumors: a university tumor institute experience

**DOI:** 10.1186/s12885-018-4648-3

**Published:** 2018-07-13

**Authors:** Andreas Toepfer, Norbert Harrasser, Maximiliane Recker, Ulrich Lenze, Florian Pohlig, Ludger Gerdesmeyer, Rüdiger von Eisenhart-Rothe

**Affiliations:** 1 Klinik für Orthopädie und Sportorthopädie Klinikum rechts der Isar der Technischen, Universität München, Ismaningerstr.22, 81675 München, Germany; 2 Wilhelm Sander-Therapieeinheit für Knochen- und Weichteilsarkome am Klinikum rechts der Isar, Ismaninger Str. 22, 81675 München, Germany; 3 Kantonspital St. Gallen, Klinik für Orthopädische Chirurgie und Traumatologie, Rorschacher Strasse 95, CH-9007 St. Gallen, Switzerland; 4 Universitätsklinikum Schleswig Holstein, Campus Kiel, Sektion für Onkologische und Rheumatologische Orthopädie in der Klinik für Unfallchirurgie, Arnold Heller Strasse, D-24105 Kiel, Germany

**Keywords:** Foot tumor, Musculoskeletal tumor, Bone sarcoma, Soft tissue sarcoma, Calcaneal bone cyst

## Abstract

**Background:**

Bone and soft tissue masses of the foot and ankle are not particularly rare but true neoplasia has to be strictly differentiated from pseudotumorous lesions. Diagnosis is often delayed as diagnostic errors are more common than in other regions. Awareness for this localization of musculoskeletal tumors is not very high and neoplasia is often not considered. The purpose of this study is to provide detailed information on the incidence and distribution patterns of foot and ankle tumors of a university tumor institute and propose a simple definition to facilitate comparison of future investigations.

**Methods:**

As part of a retrospective, single-centre study, the data of patients that were treated for foot and ankle tumors between June 1997 and December 2015 in a musculoskeletal tumor centre were analyzed regarding epidemiologic information, entity and localization. Included were all cases with a true tumor of the foot and ankle. Exclusion criteria were incomplete information on the patient or entity (e.g. histopathological diagnosis) and all pseudotumoral lesions.

**Results:**

Out of 7487 musculoskeletal tumors, 413 cases (5,52%) of tumors of the foot and ankle in 409 patients were included (215 male and 198 female patients). The average age of the affected patients was 36 ± 18y (min.3y, max.92y). Two hundred sixty-six tumors involved the bone (64%), among them 231 (87%) benign and 35 (13%) malignant. There were 147 soft tissue tumors (36%), 104 (71%) were benign, 43 (29%) malignant. The most common benign osseous tumor lesions included simple bone cysts, enchondroma and osteochondroma. By far the most common malignant bone tumor was chondrosarcoma. Common benign soft tissue tumors included pigmented villo-nodular synovitis, superifcial fibromatosis and schwannoma whereas the most common malignant members were synovial sarcoma and myxofibrosarcoma. Regarding anatomical localization, the hindfoot was affected most often.

**Conclusions:**

Knowledge of incidence and distribution patterns of foot and ankle tumors will help to correctly assess unclear masses and initiate the right steps in further diagnostics and treatment. Unawareness can lead to delayed diagnosis and inadequate treatment with serious consequences for the affected patient.

## Background

Considering the proportional mass of the foot and ankle (3%) [1], this area is affected, relatively speaking, more frequently by neoplasia than the rest of the musculoskeletal system. Data from different studies suggest that approximately 5–10% of all musculoskeletal tumors are located at the foot [2–4]. Given the rarity of musculoskeletal tumors in general, the total number of true neoplasia of the foot and ankle is small. Although the compact anatomy should facilitate early detection of tumors of the foot and ankle, the correct diagnosis is often missed due to a lack of awareness of these entities. Additionally, the malignant potential of a tumor on the foot is often underestimated [3, 5]. Despite the rarity of presentation, it is important for any specialist involved to be familiar with the diagnostic criteria and therapeutic options for these patients, as each tumor varies in its presentation, level of aggressiveness, and natural history [6].

Sarcomas are notoriously difficult to differentiate from benign lesions by clinical examination and radiographic analysis solely, and thus some malignant tumors are excised inadequately (“*unplanned resection*”). Unplanned surgical excisions of malignant tumors of the foot and ankle often result in the need for more aggressive surgery and adjuvant therapy and can adversely affect outcome and prognosis [7, 8]. Profound knowledge of the most common entities of foot and ankle tumors and their benign and malignant differential diagnoses is mandatory for a successful treatment. With appropriate diagnostic tests and treatment strategies, patients can anticipate a reasonable chance of survival and preservation of function [6]. The purpose of this study is to report the results of a retrospective, epidemiologic study of bone and soft tissue tumors of the foot and ankle in patients treated at a musculoskeletal tumor centre. Our primary aim is to describe the prevalence, demography and anatomical distribution of the tumors and compare our data with the existing literature. In this work, emphasis is laid on a standardized definition of foot and ankle tumors as many existing studies include pseudotumors and tumor-like lesions and do not use a uniform classification with regards to anatomical localization, complicating comparison enormously. This study presents an analysis of the second largest population of patients with foot and ankle tumors in the current literature so far and is intended to improve the understanding of this rare and heterogeneous pathology.

## Methods

The aim of this study is to describe the prevalence, demography and anatomical distribution of the tumors of the foot and ankle and compare our data with the existing literature. Moreover, a simple definition of foot and ankle tumors is proposed intending to facilitate future investigations.

All patients who received therapy for a tumor of the foot and ankle at a university musculoskeletal tumor centre and who subsequently were discussed at our multidisciplinary musculoskeletal tumor board for bone and soft tissue sarcomas between July 1997 and December 2015 were identified through an independent analysis of our institutional database by two different authors (AT and MR). The inclusion criteria were primary or secondary tumors that involved the foot and/or ankle, a biopsy-proven verified histological diagnosis and treatment at our institution. The exclusion criteria were insufficient data, including the lack of medical record data, imaging studies, or histologic slides, all of which contributed to a vague or inadequate identification of a tumor. All patients gave their informed consent at admission to be included in scientific studies. The investigation was approved by our institutional review board.

Foot and ankle tumors were defined according to the WHO classification of musculoskeletal tumors [9]. Thus, all tumors of undefined neoplastic nature (e.g. unicameral bone cyst, aneurysmatic bone cyst) were included and tumor-like lesions and pseudotumors (e.g. intraosseous mucoid cyst, ganglion cysts) excluded.

Regarding localization, we adapted the anatomical classification of the foot skeleton described by Ruggieri et al. to facilitate comparison of the collected data [3]. The foot skeleton can be categorized according to functional or anatomical considerations. The functional classification divides the foot into the forefoot (phalanges of the toes and metatarsals), the midfoot (lesser tarsals = cuneiform bones, navicular bone and cuboid bone) and the rear−/hindfoot (talus and calcaneus) whereas the anatomical classification distinguishes between forefoot (phalanges), the midfoot (metatarsals & lesser tarsals) and the rear−/hindfoot (talus and calcaneus).

**Fig. 1:**
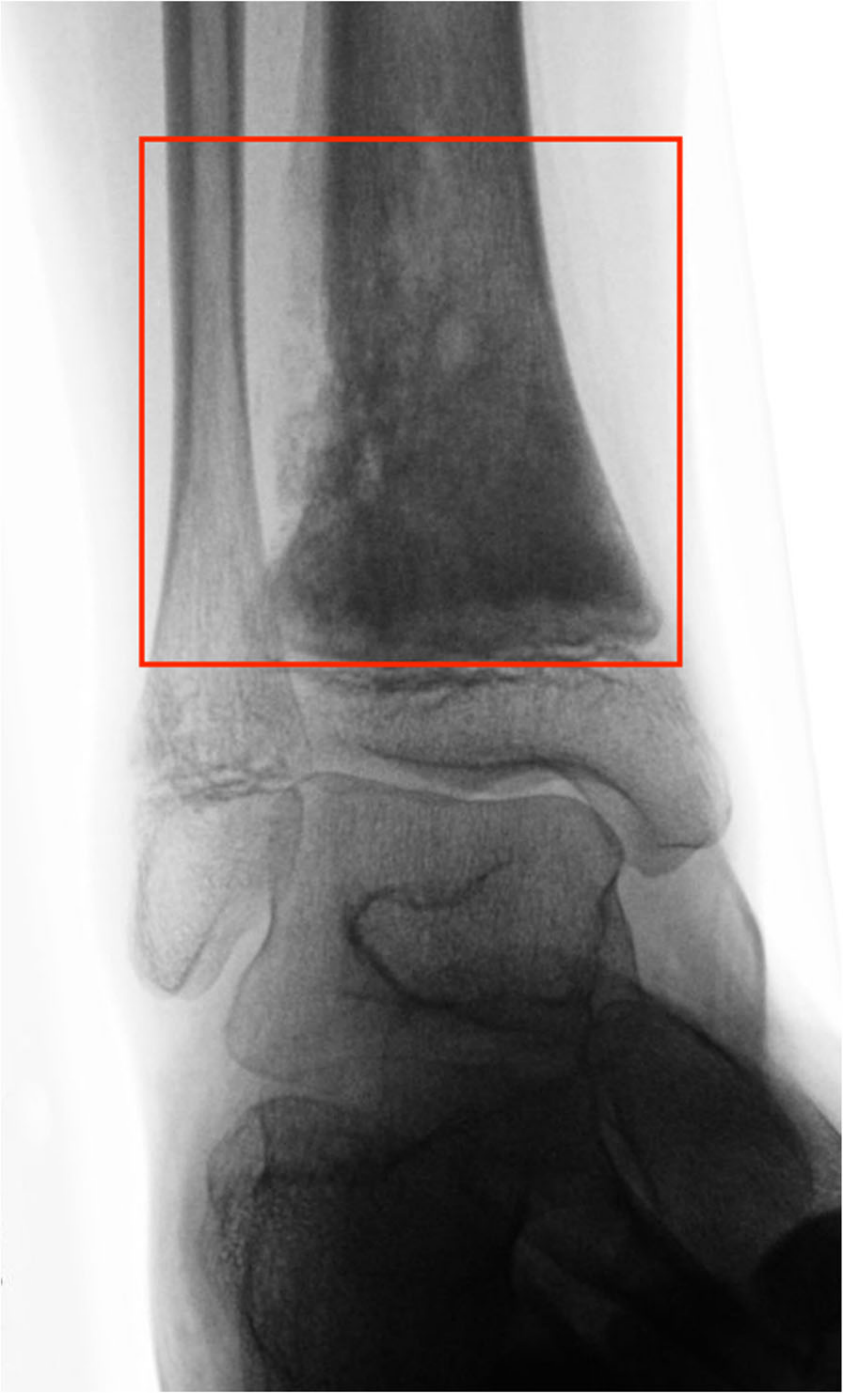
High-grade central osteosarcoma located at the distal tibial metaphysis in a 14-year old male Arab patient which fulfilled our criteria of foot and ankle tumors. The metaphysis was defined as a square the sides of which have the same length as the widest part of the growth plates. All tumors originating from the distal metaphyses of the tibia and fibula (“ankle”) were included in our study

Although Ruggieri et al. did not explicitly list the ankle as a specific anatomic region in their study [3], we feel obliged to include the distal tibia and fibula separately to avoid any misunderstanding. The upper ankle joint (talocrural articulation) represents an inherent functional part of the foot and therefore we propose to include the ankle in any study on foot tumors. Its proximal part consists of the epi-metaphysis of the distal tibia and fibula. According to the AO (*Arbeitsgemeinschaft für Osteosynthesefragen*) the metaphysis is determined by a square the sides of which have the same length as the widest part of the growth plate. In paired bones such as the tibia and fibula, both bones must be included in the square [10]. We adapted this definition and included all tumors that originated from this defined area, designated as “ankle” (Fig. 1).

It has to be noted that Kirby et al. proposed his own classification of anatomical regions for soft tissue tumors and tumor-like lesions of the foot. Here the foot is divided into five zones, corresponding to the ankle, heel, dorsum of the foot, the plantar surface of the foot, and the toes [11]. Although this classification has been used by other authors in the analysis of soft tissue lumps [12] we decided to adopt the anatomical classification of Ruggieri et al. to facilitate a direct comparison to his data. Kirby’s classification is not suitable for osseous lesions. Consequently, a stringent analysis and comparison of both osseous and soft tissue lesions would not have been feasible. In our investigation, if a soft tissue mass spread out over more than one anatomical compartment (e.g. hindfoot and midfoot) the alleged centre of the lesion was allocated to the corresponding underlying bone, respectively anatomic region.

Generally, of all benign soft tissue tumours 99% are superficial and 95% are less than 5 cm in diameter. For soft tissue sarcomas, two-thirds are deep-seated with a median diameter of 9 cm [13]. Due to its compact anatomy these findings cannot be transferred directly to the foot and ankle.

After analyzing the existing literature on this subject, the medical record review followed in large part the protocol of Ruggieri et al. [3] and was conducted by two authors (AT and MR) who gathered the following information: Patient age at treatment, sex (male/female), side (left/right), histologically verified diagnosis and anatomic localization. A review of all imaging studies, including plain radiographs, MRI and computed tomography (when available), was performed. Histological classification of the tumor, determined by biopsy, was available for all cases and reevaluated by a certified musculoskeletal pathologist. The study variables included the tissue of origin (bone or soft tissue), categorization of the lesion as benign or malignant, anatomic localization (forefoot, midfoot, hindfoot, ankle) and the histological entity. To find relevant English and non-English reports, we searched MEDLINE (US National Institutes of Health, National Library of Medicine, available at: https://www.ncbi.nlm.nih.gov/pubmed/) using the following keyword phrases: “tumor”, “bone tumor”, “soft tissue tumor”, “neoplasm” and “foot” as well as “foot and ankle tumor”. Moreover, a cross-check of all relevant references from the retrieved papers was performed to identify further studies on this subject. The data was recorded and analyzed using Excel software (Microsoft Excel 2011, Microsoft, Richmond, WA by one author (AT). Categorical variables were expressed as the frequency count and percentage of the total number of lesions in a specified category. The statistical analysis of the demographic data was performed in a descriptive manner. The mean value, standard deviation and minimum/maximum values were indicated where applicable.

## Results

### Patients, ratio of bone and soft tissue tumors and rate of malignancy

From a total of 7487 bone and soft tissue tumors treated at our musculoskeletal tumor centre and discussed in our multidisciplinary tumor board between July 1997 and December 2015, 413 (5,52%) cases of foot and ankle tumors in 409 patients matched the inclusion criteria. Two hundred nineteen tumors were located on the right distal extremity, 190 on the left and 4 bilaterally. There were 213 (52,0%) male and 196 (48,0%) female patients involved. The mean age of all patients at diagnosis was 36 ± 18 (range 3 to 91) years. Of all 413 ft and ankle tumors, 335 (81,1%) were benign and 78 (18,9%) malignant. The sex ratio for patients with benign tumors was m:f = 178:157 and for all malignant tumors, including metastases, m:w = 43:35. There were 266 bone tumors (64,4%) and 147 (35,6%) soft tissue tumors. The 266 bone tumors consisted of 231 (86,8%) benign and 35 (13,2%) malignant species (including 6 metastases).

The average age for all patients with bone tumors (m:f = 158:108) was 31 ± 17 (range 5 to 78) years, for all patients with benign bone tumors (m:f = 138:93) 29 ± 15 (range 12 to 88) years and for all patients with primary malignant bone tumors (m:f = 24:5) 44 ± 19 (range 5 to 78) years (metastases excluded). If we include metastases to all malignant bone tumors the age distribution was 46 ± 20 (range 5 to 78) and the sex ratio m:f = 26:9. For the metastases alone, age distribution was 66 ± 7 (range 58 to 75) years and the sex ratio m:f = 2:4.

Out of 147 patients with soft tissue tumors (m:f = 57:90) there were 104 (70,7%) benign (m:f = 40:64) and 43 (29,3%) malignant (m:f = 17:26) cases. The average age of all soft tissue tumors was 45 ± 18 (range 3 to 92) years, for all benign soft tissue tumors 40 ± 16 (range 8 to 86) years and for all malignant soft tissue tumors 57 ± 18 (range 3 to 92) years. There were no soft tissue metastases.

**Fig. 2:**
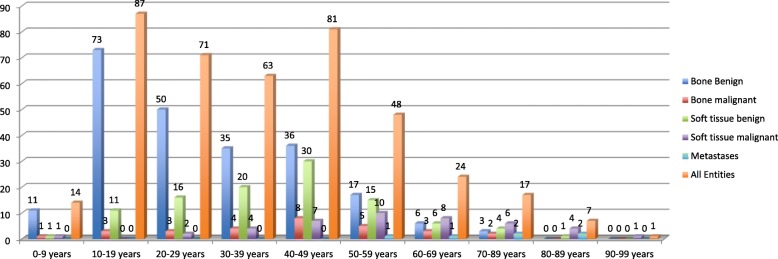
Age distribution for benign and malignant bone and soft tissue tumors. Metastases are shown separately

A histogram illustrating the distribution of patient age is provided with Fig. 2.

### Tumor entities

Altogether, 49 different tumor entities were identified, subtypes (e.g. exophytic chondrosarcoma) and metastases, pseudotumors and tumor-like lesions not counted. The top five entities of each category will be shortly listed in the following paragraphs, for more detailed information on other tumor entities, please see Tables 1, 2, 3 and 4.

**Table 1:** Benign bone tumors

	Forefoot	Midfoot	Hindfoot	Ankle	total	male	female
Unicameral Bone Cyst	1	1	42	6	50	34	16
Enchondroma	30	6	2	4	42	25	17
Osteochondroma	8	3	4	13	28	17	11
ABC	1	8	12	6	27	15	12
Intraosseous Lipoma	0	0	21	0	21	14	7
Giantcell Tumor	0	8	2	7	17	5	12
Osteoidosteoma	2	2	4	6	14	12	2
NOF	0	0	0	13	13	6	7
Chondroma	4	2	1	1	8	5	3
Chondroblastoma	0	1	2	0	3	3	0
Chondromyxofibroma	0	0	1	1	2	1	1
Fibrous Dysplasia	0	0	0	2	2	0	2
intraoss.Hemangioma	0	0	1	1	2	0	2
Osteoblastoma	0	0	1	0	1	0	1
Osteofibroma	0	0	0	1	1	1	0
total	46	31	93	61	231	138	93

Benign bone tumors with entities, localization and sex distribution

**Table 2:** Malignant bone tumors

	Forefoot	Midfoot	Hindfoot	Ankle	total	male	female
Chondrosarcoma	4	3	6	4	17	14	3
Osteosarcoma	0	1	1	4	6	4	2
Ewing Sarcoma	0	2	2	1	5	5	0
Fibrosarcoma	1	0	0	0	1	1	0
Metastases	2	2	2	0	6	2	4
total	7	8	11	9	35	26	9

Malignant bone tumors with entities, localization and sex distribution

**Table 3:** Benign soft tissue tumors

	Forefoot	Midfoot	Hindfoot	Ankle	total	male	female
Hemangioma	8	8	4	7	27	11	18
PVNS	6	5	1	6	18	4	14
Fibromatosis	0	15	0	0	15	8	7
Neurinoma	1	0	8	2	11	5	6
Angiomyoma	2	0	0	6	8	4	4
Lipoma	0	1	2	5	8	2	6
Fibroma of tendon sheath	1	1	0	3	5	1	4
Erdheim-Chester disease	0	0	0	2	2	1	0
Glomangioma	2	0	0	0	2	2	0
Lymphangioma	1	0	0	1	2	0	2
Benign fibrous histicytoma	0	0	0	1	1	0	1
Fibrolipoma	1	0	0	0	1	0	1
Desmoplastic Fibroblastoma	0	0	1	0	1	1	0
Calcifying aponeurotic Fibroma	1	0	0	0	1	0	1
Myxoma	0	0	0	1	1	0	1
Poroma	1	0	0	0	1	0	1
total	24	30	16	34	104	40	66

Benign soft tissue tumors with entities, localization and sex distribution

**Table 4:** Malignant soft tissue tumors

	Forefoot	Midfoot	Hindfoot	Ankle	Total	male	female
Synovial sarcoma	1	5	1	3	10	5	5
Myxofibrosarcoma	1	3	0	4	8	3	5
Malignant Melanoma	4	3	1	0	8	5	3
Leiomyosarcoma	0	0	1	3	4	1	3
Fibrosarcoma	0	1	0	1	2	0	2
Lymphoma	0	1	1	0	2	0	2
Epithelioid Sarcoma	0	2	0	0	2	1	1
Angiosarcoma	0	0	0	1	1	1	0
MPNST	0	1	0	0	1	0	1
Myoepithelial Carcinoma	0	0	1	0	1	0	1
NOS Sarcoma	0	0	0	1	1	0	1
Pleomorphic Sarcoma	0	0	0	1	1	1	0
Liposarcoma	0	1	0	0	1	0	1
M.Bowen	1	0	0	0	1	0	1
Total	7	17	5	14	43	17	26

Malignant soft tissue tumors with entities, localization and sex distribution

### Benign bone tumors

For a total of 231 benign bone tumors (231/413, 55,9%), there were 15 different entities in which the top five accounted for 72,7% (*n* = 168) of all 231 cases (Table 1). Accordingly, the remaining ten tumor entities summed up to only 27,3% (*n* = 63) of all benign bone tumors. The most prevalent benign bone lesions were unicameral bone cyst which accounted for 50 (21,6%) of the 231 non-malignant bone tumors, followed, in descending order, by enchondroma (*n* = 42, 18,2%), osteochondroma (*n* = 28, 12,1%), aneurysmatic bone cyst (ABC, *n* = 27, 11,6%) and intraosseous lipoma (IOL, *n* = 21, 9,0%).

### Malignant bone tumors

Thirty-five malignant bone tumors corresponded to 8,5% of the entire collective, and consisted of four different types of primary bone malignancies (sarcomas), and three different types of metastases (Table 2). Chondrosarcoma (*n* = 17, 48,6%), osteosarcoma (*n* = 6, 17,1%), Ewing sarcoma (*n* = 5, 14,3%) and fibrosarcoma (*n* = 1, 2,8%) accounted for the bone sarcomas. Breast- (*n* = 4), prostate- (*n* = 1) and gastric cancer (*n* = 1) comprised of the three different types of metastases (*n* = 6, 17,1%).

### Benign soft tissue tumors

Benign soft tissue tumors accounted for 25,2% of all included tumors. Sixteen different entities composed the spectrum of the 104 benign soft tissue tumors (Table 3). Hemangioma was the most common entity in this category (*n* = 27, 25,9%), followed by pigmented villo-nodular synovitis (PVNS, *n* = 18, 17,3%), superficial fibromatosis (Ledderhose disease, *n* = 15, 14,4%) and neurinoma/schwannoma (*n* = 11, 10,5%). The fifth spot was shared by angiomyoma/angioleiomyoma and lipoma, both with 8 cases (7,7%), respectively. It is worth mentioning, that the majority of entities (10/16, 62,5%) contributed five or less cases to the total number of 104 benign soft tissue tumors.

### Malignant soft tissue tumors

Of the 43 (10,4%) malignant soft tissue tumors out of all 413 ft and ankle tumors, the most prevalent types were synovial sarcoma (*n* = 10, 23,2%), myxofibrosarcoma (*n* = 8, 18,6%), malignant melanoma (*n* = 8, 18,6%) and leiomyosarcoma (*n* = 4, 9,3%). The fifth most common malignant soft tissue tumors were fibrosarcoma, lymphoma (malignant lymphoma in soft tissue) and epithelioid sarcoma with two cases (4,6%), respectively. Eleven out of 14 different entities in this category contributed to less than five cases each, demonstrating the great diversity of potential diagnoses once more (Table 4).

### Sites of involvement

Table 5 provides an overview of the distribution patterns regarding anatomic localization. Benign bone tumors showed a clear prevalence for the hindfoot (*n* = 93, 40,2%), followed by the ankle (*n* = 61, 26,4%), the forefoot (*n* = 46, 19,9%) and, lastly, the midfoot with 31 cases (13,4%).

**Table 5:** Overview of the distribution patterns of all benign and malignant foot and ankle tumors

	Forefoot	Midfoot	Hindfoot	Ankle	total	male	female	right	left	bilateral
bone benign	46	31	93	61	231	138	93	129	101	1
bone malignant	5	11	10	9	35	20	15	22	13	–
bone total	51	42	103	70	266	158	108	149	114	1
soft tissue benign	24	30	16	34	104	40	64	51	40	3
soft tissue malignant	7	17	5	14	43	17	26	22	21	–
soft tissue total	31	47	21	48	147	57	90	73	71	3
benign tumors	70	61	109	95	335	178	157	180	151	4
malignant tumors	12	28	15	23	78	43	35	44	34	–
all tumors	82	89	124	118	413	221	192	224	185	4

Overview of the distribution patterns of all benign and malignant foot and ankle tumors regarding anatomic localization

For malignant bone tumors, the midfoot (*n* = 11, 31,4%), hindfoot (*n* = 10, 28,6%) and the ankle (*n* = 9, 25,7%) were almost equally affected. The forefoot showed 5 cases of malignant bone tumors (14,3%). Overall, bone tumors were most commonly localized at the hindfoot (*n* = 103, 38,7%).

Benign soft tissue tumors distributed as follows: Ankle area (over the epi-metaphysis of the distal tibia and fibula, *n* = 34, 32,7%), midfoot (*n* = 30, 28,8%), forefoot (*n* = 24, 23,1%) and hindfoot (*n* = 16, 15,4%). Malignant soft tissue tumors were most commonly situated at the midfoot (*n* = 17, 39,5%), over the ankle (*n* = 14, 32,5%), the forefoot (*n* = 7, 16,3%) and the hindfoot (*n* = 5, 11,7%). Overall, soft tissue tumors of the foot and ankle were most commonly and almost equally distributed between the area over the ankle (*n* = 48, 32,6%) and the midfoot (*n* = 47, 31,9%).

The entirety of foot and ankle tumors showed a more balanced distribution over the four anatomic compartments (Table 5): 124 cases were localized at the hindfoot (30,0%), 118 cases at the ankle (28,6%), 89 at the midfoot (21,5) and 82 at the forefoot (19,9%). 54,2% of all tumors were located on the right foot and ankle, 44,8% left and 1% bilaterally. All four cases of bilateral involvement included benign tumors (1× multiple exostoses, 1× Erdheim-Chester disease [14], 2× plantar fibromatosis).

## Discussion

Considering the proportional mass of the foot and ankle region, it is disproportionately affected by musculoskeletal tumors: The segment weight of a single human foot as percent of the total body weight is specified as 1,45 ± 0,126%, including the lateral malleolus [1, 15, 16]. The literature shows that 5–10% of musculoskeletal tumors involve the foot and ankle [2, 4]. As reported by Kransdorf et al., the American Forces Institute of Pathology series on 39,179 soft tissue tumors found that 8% of all benign and 5% of malignant soft tissue tumors in the body occur in the foot and ankle [17, 18].

In 1997, Ozdemir et al. reported 1786 bone and soft tissue tumors of which 196 (10.9%) involved the foot and ankle. Of these 87.2% were benign and the remaining 12.8% were malignant. Of these 87.2% were benign and the remaining 12.8% were malignant [2].

In a review of 2660 musculoskeletal tumors treated at a musculoskeletal tumor referral centre, Chou et al. found 153 cases (5.75%) located in the foot and ankle, with 60.8% benign lesions [4].

Many authors fail to provide accurate description of patient selection, for example Pollandt et al. noted 4.5% of all musculoskeletal tumors affecting the foot and ankle region, but failed to note the overall number of tumors [19]. In our study, 413 out of 7847 tumors treated over a period of 18,5 years were located at the foot and ankle, accounting for 5,52% of all tumors. Over the course of the investigation period we encountered an average of 22,3 ft and ankle tumors per year, although annual numbers continuously increased over the last years (*n* = 41 in 2014 and *n* = 50 in 2015). Only Ruggieri et al. describe a higher incidence from a single centre analysis (*n* = 68,8 per year, pseudotumors included). In contrast to many other authors, including some of the largest studies on this subject [3, 4, 19], we excluded pseudotumorous lesions like ganglion cysts or inclusion cysts. Although pseudotumors make up a significant portion of all tumorous lesions of the foot and ankle we intentionally decided to exclude pseudotumorous masses to make future comparative studies more precise and manageable. Nevertheless, it is strongly advised to include pseudotumorous lumps and bumps in the differential diagnosis to avoid overtreatment.

Malignant tumors were found in 78 cases (18,8%) in the present study. Soft tissue tumors demonstrated a higher rate of malignancy (29.2%) in comparison to bone lesions (13.1%). In general, benign mesenchymal tumours outnumber sarcomas by a factor of at least 100 and soft tissue sarcomas are approx. Four times more common than bone sarcomas and [13]. In Ruggieri’s cohort, the rate of malignancy was higher in all subgroups: 20,6% for bone tumors, 51,8% for soft tissue tumors and 25,6% in the total cohort. The proportional amount of soft tissue tumors was lower (16,1%) but the total numbers were higher for all subgroups (bone tumors: *n* = 981, soft tissue tumors: *n* = 189) [3].

Malignant bone tumors demonstrated an even anatomical distribution pattern in our series whereas benign bone tumors showed a strong predilection for the hindfoot (40,25%). This may be contributed by the fact that two of the most common benign bone tumors of the foot and ankle, UBC (*n* = 50) and IOL (*n* = 21) accounted for 30,7% of all benign bone tumors and are found almost exclusively at the calcaneus. A detailed comparison to the existing studies focused on tumors of the foot and ankle is provided in Table 6. While a direct comparison between most publications is difficult due to heterogeneous study protocols (e.g. inclusion criteria, definitions of tumor and anatomic localization), it becomes clear that foot and ankle tumors show a great diversity. Many entities, in particular malignant lesions, do not present with consistent patterns of anatomic distribution within the foot and ankle. This is why the existing data as well as our own results cannot be used like a map of where to find which tumor entity rather than emphasizing the fact that any suspicious lump or bump in the foot and ankle region should be consider a tumorous process unless proven otherwise [20, 21]. Of note is that a variety of very rare tumors (e.g. epithelioid sarcoma) show a strong predilection for the foot and can imitate less aggressive, benign lesions [22, 23]. Benign tumors and tumor-like lesions are much more common than malignant tumors and soft tissue tumors are generally more common than bone tumors [9]. However, three of the largest current studies, this one included, seem to indicate a different ratio for foot and ankle tumors, with the total number of bone tumors clearly exceeding their soft tissue counterparts [3, 24].

**Table 6:** Literature overview

Year	Number of cases	Author	Journal	Bone tumor (benign/malignant)	Soft tissue tumor (benign/malignant)	Overall (benign/malignant)	Period of time in (years) & tumors per year
1989	83* (ganglion cysts)	Kirby [11]	JBJS Am	–	83 (72 / 11)	72 (87%) / 11 (13%)	5y, 16,6 / year
1989	255	Murari [26]	Foot Ankle Int	255 (213 / 42)	–	213 (83%) / 42 (16%)	16y 21,2 / year
1994	33* (inlcusion cysts)	Chou [32]	Foot Ankle Int	14 (6 / 8)	19 (15 / 4)	21 (63%) / 11 (37%)	14y 2,3 / years
1996	26	Sarkar [33]	Foot Ankle Surg	20 (15 / 5)	6 (3 / 3)	15 (58%) / 11 (42%)	13y 2,0 / years
1997	196	Ozdemir [2]	J Foot Ankle Surg	136 (130 / 6)	60 (41 / 19)	171 (87%) / 25 (13%)	12y 16,3 / years
2002	62	Kinoshita [34]	Orthop Proceedings	34 (30 / 4)	28 (25 / 3)	55 (89%) / 7 (11%)	24y 2,6 / years
2002	83	Kinoshita [35]	J Orthop Surg	36 (33 / 3)	47 (42 / 5)	75 (90%) / 8 (10%)	26a 3,2/ years
2003	367* (ganglion cysts)	Pollandt [19]	Z Orthop Grenz	367 (292 / 75)	–	292 (80%) / 75 (20%)	n.a.
2005	204	Buchner [24]	Der Chirurg	153 (129 / 24)	51 (34 / 17)	163 (80%) / 41 (20%)	17y 12 / year
2007	166	Delgado [36]	Acta Orthop Mex	81 n.a.	79 n.a.	n.a.	10y 16,6 / year
2009	153 * (ganglion cysts)	Chou [4]	Foot Ankle Int	73 (56 / 17)	80 (42 / 38)	93 (61%) / 60 (39%)	20y 7,6 / year
2010	75*	Hofstätter [37]	WMW	36 (29 / 7)	39 (28 / 11)	57 (76%) / 18 (24%)	22y 3,4 / year
2012	170*	Li [38]	Chin J Orthop	51 n.a.	119 n.a.	n.a.	25y 6,8 / year
2013	72	Azevedo [39]	J Foot Ankle Surg	9 (7 / 2)	63 (49 / 14)	56 (78%) / 16 (22%)	10y 7,2 / year
2014	1170* (ganglion cysts)	Ruggieri [3]	J Foot Ankle Surg	981 (779 / 202)	189 (91 / 98)	870 (74%) / 300 (26%)	17y 68,8 / year
2014	67* (ganglion cysts)	Kim [40]	Int J BioScie	13 (12 / 1)	54 (49 / 5)	61 (91%) / 6 (9%)	7y 9,5 / year
2017	413	Toepfer		266 (231 / 35)	147 (104 /43)	335 (81%) 78 (19%)	18,5y 22,3 / year

Comparison of the existing literature. All studies marked with an asterisk (*) included pseudotumorous lesions

Ruggieri et al. found at least 16 different entities of bone tumors in his patients [3] and we identified 15 different entities in our investigation. Soft tissue tumors can show an even broader range of entities than bone tumors. Our own study revealed 30 different entities for 147 soft tissue tumors.

Discrepancies between the radiological and definitive histological diagnosis are not uncommon for foot and ankle tumors [25]. Both primary malignant bone and soft tissue tumors such as chondrosarcoma or synovial sarcoma as well as metastases represent relevant differential diagnoses of unknown bone and soft tissue lesions. Some of the most common osseous lesions of the foot and ankle, as shown in several studies, are unicameral bone cyst, enchondroma and osteochondroma [2–4, 6, 26, 27]. For these entities, diagnostics are often unambiguous and therapy is straight forward [28, 29].

**Fig. 3:**
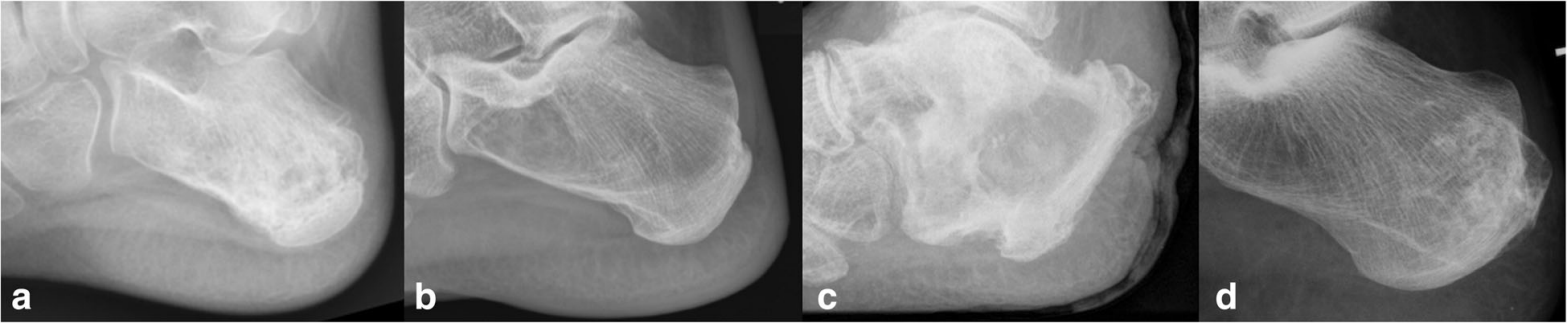
Osteolytic lesions of the calcaneus with different radiographic appearance and varying aggressive behaviour: (a) Ewing sarcoma in a 31-year old male patient, (b) simple (calcaneal) bone cyst in a 11-year old male patient, (c) secondary squamous cell carcinoma based on chronic osteomyelitis in a 82-year old male patient and (d) low-grade chondrosarcoma in a 45-year old female

The highly variable clinical presentation of malignant bone tumors about the foot and ankle might explain the high number of delayed or missed diagnoses (Fig. 3) [30]. The delay in diagnosis of these tumors is significantly longer than that of equivalent tumors at other skeletal sites [5, 31].

There are a number of limitations to our study that could have influenced our conclusions: Study design and impact limitations include that all data were obtained from a single centre. The cases referred to our musculoskeletal tumor referral centre are often specific and might be more advanced or symptomatic. The vast majority of cases in our investigation were treated surgically. Thus, benign and asymptomatic cases that were not discussed in our multidisciplinary musculoskeletal tumor board may have been missed. Most patients included in this study are of caucasian origin. Our findings may not unrestrictedly translate to patients of other ethnicities. Nevertheless, our results might still be widely applicable and help to raise awareness for this rare pathology.

Statistical and data limitations include that some tumor entities are very rare, so that large numbers are difficult to obtain even over a long period of time (especially for malignant tumors), possibly underpowering our results. Still, 78 malignant tumors of the foot and ankle are more than most other studies were able to report. Only Ruggieri et al. presented a larger number of malignancy at the foot and ankle in his single centre investigation [3]. As previously stated by Chou et al., the low incidence of foot and ankle tumors, combined with the large number of possible histologic diagnoses, makes it challenging to accumulate enough patients to make reliable conclusions about specific diagnoses in this anatomic region [4].

## Conclusions

Bone and soft tissue lesions resulting from trauma, degeneration, inflammation or deformity are not particularly rare at the foot and ankle but have to be differentiated from lesions of tumorous etiology. The absolute number of foot and ankle tumors is relatively small but the diversity of potential entities is profound. For a malignant neoplastic disease, early diagnosis and proper management are key factors in increasing the life expectancy and functional outcome of these patients [3, 5].

Thus, any physician approaching a patient with a suspicious lesion of the foot should always include a tumorous process in the differential diagnosis.

Statistics on tumors of the entire musculoskeletal system cannot uncritically be translated to the foot and ankle region. The existing data on foot and ankle tumors as well as our own results cannot be used like a map of where to find which tumor entity rather than emphasizing the fact that any suspicious lump or bump in the foot and ankle region should be consider a tumorous process unless proven otherwise.

Knowledge of potential tumor entities and distribution patterns, as provided by this study, can help to improve the understanding of the heterogeneous pathology of foot and ankle tumors and, consequently, ameliorate the therapeutic success. The findings of this study show how heterogeneous the diagnosis “foot tumor” really is. Accordingly, foot and ankle tumors must be analyzed very carefully in clinical practice.

## Abbreviations

ABC: Aneurysmatic bone cyst
IOL: Intra-osseous lipoma
MRI: Magnet resonance imaging
PVNS: Pigmented villo-nodular synovitis
UBC: Unicameral bone cyst
